# Outbreak Investigation of Marek’s Disease in Chickens in Central and Western Ethiopia

**DOI:** 10.1155/vmi/1760044

**Published:** 2026-09-21

**Authors:** Yobsan Tamiru, Habtamu Mekonnen, Bayeta Senbata, Demessa Negessu, Abde Aliyi, Begna Bulcha, Abriham Kebede, Waktola Tadesse, Debela Abdeta

**Affiliations:** ^1^ School of Veterinary Medicine, Wallaga University, Nekemte, Ethiopia, wollegauniversity.edu.et; ^2^ Livestock Office, Hurumu, Ethiopia; ^3^ Virus Isolation and Molecular Laboratory, Animal Health Institute, Sebeta, Ethiopia, ahi.org; ^4^ College of Veterinary Medicine and Agriculture, Addis Ababa University, Bishoftu, Ethiopia, aau.edu.et

**Keywords:** Ethiopia, Marek’s disease, molecular detection, outbreak

## Abstract

**Background:**

Marek’s Disease (MD) is an economically devastating poultry disease that causes substantial livestock losses and drives up overall health management costs. There was no study conducted on isolation and molecular detection of Marek’s disease virus (MDV) in extensively managed local breed chickens in some parts of Central and Western Ethiopia.

**Methods:**

The present study was conducted from September 2021 to October 2022 with the aim of isolation and molecular detection of MDV suspected from outbreak cases in chickens in Central and Western Ethiopia. A total of 94 pooled samples were collected from clinically suspected chickens from four areas, namely, Mako, Guto Gida, Holeta, and Sebeta subcity. Samples were collected from chickens in both intensive and extensive production systems, but nonvaccinated birds against MDV were obtained only from the extensive system.

**Result:**

From the clinical samples tested based on quantitative real‐time PCR, 75 (79.78%; 75/94) tested positive for meq gene of the virus. Of these, 36.17% (34/94) of the positive samples were obtained from the feather (34/41) and the rest (43.62%; 41/94) were obtained from postmortem tissue (41/53) samples. From the overall prevalence of 79.78% (75/94), the highest score was recorded in Holeta town (95.65%), followed by Mako District (88.89%), Sebeta Subcity (62.5%), and Guto Gida District (57.89%). Though there are confounding factors such as vaccination status, significantly (*p* = 0.04) higher real‐time PCR positive results were obtained in exotic breeds. From 10 pooled liver samples, all samples (100%) tested positive for the virus using real‐time PCR. Similarly, all examined organs showed complete positivity: kidney (10/10, 100%) and spleen (10/10, 100%). However, from six pooled samples of proventriculus samples, only one (1/6, 16.67%) sample was positive for the virus, whereas 34 (82.9%) pooled feather follicles from clinically sick chickens were positive for MDV1. From a total of 75 real‐time PCR positive samples inoculated into DF‐1 cells for virus isolation, 45 (60%) of them were able to show cytopathic effects (CPEs) on the cell line up to the third passage. The seven (40%) feather samples remained negative against the test until the third passage.

**Conclusion:**

This study provides critical insights into the isolation and molecular detection of MDV in chickens from Central and Western Ethiopia, highlighting its significant impact on poultry health and productivity. Detailed genetic characterization of the virus and differentiation of the vaccinal strain from the natural infection in exotic breeds are necessary to have effective prevention and control. Experimental studies are also needed to evaluate differences in susceptibility to MDV among local and exotic poultry breeds.

## 1. Introduction

Poultry production has a tremendous role in the economy of developing countries like Ethiopia. It has become a significant component of agricultural sector in Ethiopia, contributing to food production and house food security [1, 2]. To promote this, the Ministry of Agriculture in the country has developed a national plan called bounty of basket and more specifically put its focus toward development of small and commercial scales of poultry enterprises with the aim of increasing meat and egg productions and then ensuring food security (Ethiopia National Poultry Development Strategy, 2022–2031). Despite these all plans, the prevalence of various poultry diseases poses significant challenges to the sector [3]. Among viral diseases affecting chickens, Marek’s disease (MD) is one of most economically important, causing substantial morbidity and mortality rates in both backyard and commercial poultry farms [4, 5].

MD is a highly oncogenic, lymphoproliferative, and neuropathic viral disease of gallinaceous birds that severely impacts the chicken production sector due to reduced egg production and carcass rejection [6–8]. This disease significantly affects poultry, thereby impacting the private sector and national economy by reducing production and productivity and causing high fatality rate. In susceptible flocks, it can lead to large levels of mortality in vulnerable birds and substantial economic losses due to an overall cost of health management [9, 10].

MD is a major obstacle to the development of the poultry industry and a significant deterrent to investment in this sector [11, 12]. The disease has been introduced into Ethiopia through exotic breeds and is now a growing concern for poultry farming systems. It is commonly found in Ethiopia and contributes to substantial health and mortality challenges in chickens across diverse production systems [13]. A study on MD outbreaks in a commercial poultry farm in Central Ethiopia reported a 46% mortality rate, with similar rates found in local chicken breeds, indicating the fatality of MD even in indigenous breeds [5]. Conducting genetic analyses of virus isolates and developing effective vaccines using local strains are essential [14]. Although it poses a serious challenge to the poultry sector, limited research has been conducted on the current status of MD, particularly its molecular characteristics in Ethiopia [13, 15]. Majority of the studies so far conducted in Ethiopia were on intensively managed chickens. No research has specifically addressed the current molecular status of MD in extensively managed and unvaccinated local breed chickens particularly in some parts of Central and Western Oromia, Ethiopia, despite its significant impact in the area. Some of the research studies conducted on local breeds mainly focus on serological status of the disease [16, 17]. Therefore, the objectives of this study include investigation of the outbreak and isolation and molecular detection of Marek’s disease virus (MDV) in both intensively managed exotic breed chickens and extensively managed local breed chickens in Central and Western Ethiopia.

## 2. Materials and Methods

### 2.1. Description of the Study Area

**FIGURE 1:**
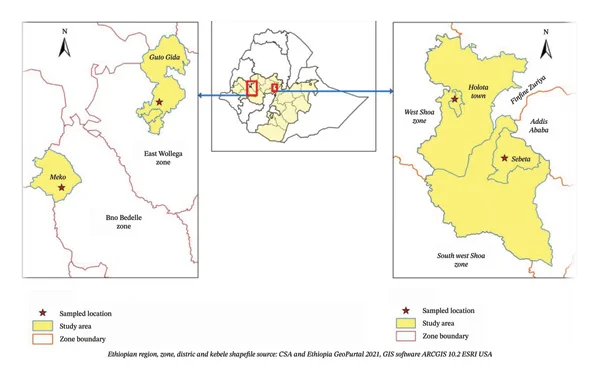
Maps of Ethiopia showing the study areas.

This study was conducted in MD outbreak areas, particularly in Mako from Buno Bedelle, Holeta from West Shewa, Sebeta Subcity from Sheger City, and Guto Gida District from East Wollega Zone (Figure 1) from September 2021 to October 2022.

### 2.2. Study Animal

The study evaluated chickens raised under both intensive and extensive production system that were affected by MD outbreaks. Chickens displaying clinical symptoms indicative of MD such as depression, weight loss, blindness, and partial leg paralysis were included in this study. All age and sex groups were part of the investigation. A total of 94 pooled samples were collected from 38 chickens showing signs of illness. These included 36 samples from Mako District, 23 from Holeta, 16 from Sebeta Subcity, and 19 from Guto Gida. Specifically, 8 pooled tissues of ischiatic nerves (each with three tissues), 41 pooled feather samples (each with four to five feather follicles), and 10 pooled samples each from the liver, kidney, and spleen tissues (each with three tissues), along with 6 pools of proventriculus, were collected. Totally, 41 pooled feathers and 53 pooled tissue samples were collected. The number of examined chickens based on their breeds is depicted in Table 1.

**TABLE 1:** Number of examined chickens based on breed.

Area	No. of chickens examined	
	Local breed	Exotic breed
Mako	5	7
Guto Gida	4	4
Holeta	4	6
Sebeta Subcity	2	6
Total	**15**	**23**

*Note:* Bold values are the values that depend on the type of breed (local vs exotic breed).

### 2.3. Study Design and Sampling Method

An outbreak case‐based study was conducted based on reports of active MDV outbreaks to collect samples from clinically affected chickens. The extensively managed local breed chickens had no history of MDV vaccination, as confirmed by local veterinary professionals, whereas those chickens under intensive management system were vaccinated against MDV. Samples were randomly collected from affected chickens for virus isolation and molecular detection at the Animal Health Institute in Sebeta, Ethiopia. The outbreak cases were identified based on information collected from regional laboratories in the Oromia Regional State and from professionals working in the study area through phone communication.

### 2.4. Sample Collection and Transportation

Sample collections were performed based on the guideline recommended by OIE [18]. Information on the disease specifics and vaccination history was gathered during sample collection. Clinically suspected chicken samples were individually collected and pooled into groups of three (for tissues) to four (for feathers) based on type and location after collection. These samples were placed in tubes with viral transport media (VTM, pH 7.4) containing phosphate‐buffered solution (PBS) and antibiotics, labeled, and transported under cold conditions to the Animal Health Institute in Sebeta.

### 2.5. Clinical Examination and Postmortem Examination

Clinical signs (paralysis of the legs (unilateral) and wings, with enlargement of peripheral nerves and gray colored iris) in diseased chickens were closely monitored. Clinically affected chickens were identified and sampled, with feather samples taken from clinically ill chickens. All examinations were performed on birds that had died with the case within 8–12 h at the time of sample collection. Characteristic lesions (enlargement of peripheral nerves and visceral tumors) in various organs and tissues were examined and prepared.

### 2.6. Laboratory Investigation

#### 2.6.1. Sample Preparation

Samples were pooled by type and study area. Tissue and feather follicle samples were minced using sterile tools and ground with sterile sand in a mortar and pestle. A 10% (w/v) suspension was made using PBS mixed with 2% antibiotic‐antimycotic solution. The mixture was then centrifuged at 3000 rpm for 10 min. Then, the clear liquid (supernatant) was carefully filtered and stored at −20°C until it was ready to be used for inoculation.

### 2.7. DNA Extraction

The samples were first thawed at room temperature, and then DNA was extracted using the QIAamp DNA Mini Kit (250), following the instructions provided by the manufacturer. Tissue suspensions (200 μL) were mixed with proteinase K and Buffer AL, incubated at 56°C for 30 min, and then mixed with ethanol. The mixtures were processed through QIAmp spin columns with sequential washes using Buffers AW1 and AW2, and finally, Buffer AE was added to elute the DNA. Feather follicle samples were processed in a biosafety cabinet and treated similarly with a longer incubation at 56°C for 30 min (Appendix II).

### 2.8. Real‐Time Polymerase Chain Reaction

All the samples collected from 38 chickens showing signs of the disease were tested for the presence of the MDV gene using quantitative real‐time PCR. Real‐time PCR tests were done according to manufacturer’s instructions, to detect MDV specific meq gene by using QIAamp DNA Mini Kit (250) (QIAGEN) [19]. Using an Applied Biosystems 7500 Fast Real‐Time PCR thermal cycler, the master mix for each 25 μL reaction was prepared with the following components: 10 μL of Platinum PCR mix, 0.6 μL of forward MDV‐1 primer (5′‐ GGA‐GCC‐GGA‐GAG‐GCT‐TTA‐TC‐3′), 0.6 μL of reverse primer (MDV‐1 (5′‐GGA‐GCC‐GGA‐GAG‐GCT‐TTA‐TC‐3′), 0.6 μL of reverse primer MDV1OPR (5′‐ATC‐TGG‐CCC‐GAA‐TAC‐AGG‐GAA‐3′), 0.8 μL of probe (5′‐CGTCTTACCGAGGATCCCGAACAGG‐3′), 1 μL of BSA (5 mg/mL), and 2 μL of DNase‐ and RNase‐free water (QIAGEN). MDV known positive isolate (5 μL) and DNAase‐free water (5 μL) were used as positive and negative control, respectively. All the reagents were combined in a single reaction tube to create a master mix, ensuring even distribution (Appendixes III and IV). The tube was then sealed to prevent evaporation during the process. The PCR was carried out with the following protocol: an initial denaturation at 95°C for 15 min, followed by 45 cycles consisting of denaturation at 95°C for 15 s, annealing at 60°C for 15 s, and extension at 72°C for 25 s [18, 20].

#### 2.8.1. Virus Culture and Isolation

Douglas Foster 1 (DF‐1, chicken fibroblast cell line derived from East Lansing Line (ELL‐0) embryonic fibroblasts) was used for isolation (Appendix V). It was employed on PCR positive samples. All the homogenized samples were spun in a centrifuge at 3000 rpm for 20 min at 4°C. After that, the clear supernatants were collected and used for virus culture. To assess the presence of the virus, 0.1 mL of the clarified tissue homogenate was transferred onto confluent DF‐1 cell monolayers maintained in 25 cm^2^ tissue culture flask, following the cell culture standard procedure described by Freshney [21]. The inoculated cells were placed in an incubator set to 37°C, with 5% CO_2_ and 100% humidity. They were gently shaken every 15 min over the course of an hour to help the virus attach to the cells. After this incubation period, the virus‐containing solution was carefully removed, and the infected cells were washed with PBS to remove any unabsorbed virus particles. Then, 0.4 mL of GMEM supplemented with 2% calf serum was added. Following inoculation, the DF‐1 cell cultures were maintained at 37°C in a humidified incubator supplied with 5% CO_2_ for 5 days. The cultures were examined each day using an inverted microscope to monitor the development of cytopathic changes associated with viral replication. Samples that showed no detectable cytopathic effects (CPEs) were subjected to two additional blind passages, resulting in a total of three passages. Cultures remaining free of CPEs after the third passage were considered negative for the presence of infectious virus.

### 2.9. Ethical Approval and Informed Consent

All samples were collected exclusively from chickens that had died naturally from the condition within the past 12 h, ensuring that no live animals were harmed, handled, or monitored for the purpose of this study. Consequently, standard live‐animal handling, weight checks, or health monitoring procedures were not applicable to this protocol. The research design and sampling plan were reviewed and approved by the Animal Welfare and Research Ethics Review Committee (AWRERC) of Wollega University, under the ethical approval number Vetm 21/8/2022‐25/8/2022. The full study protocol submitted to and approved by the ethics committee has been provided as depicted in Appendix 1. Every step of this investigation was carried out in accordance with recognized ethical guidelines at the institutional, national, and international levels. Additionally, explicit informed consent was obtained from the flock owners before performing postmortem examinations on the deceased chickens, in full respect of institutional ethical standards and poultry owner rights.

### 2.10. Data Management and Analysis

All data from the laboratory tests were carefully coded and entered into Microsoft Excel for organization and review. Before analysis, the data were thoroughly checked for accuracy. Statistical analysis was carried out using R software Version 4.0.3 [22]. Results were summarized with descriptive statistics, including percentages. The real‐time positivity rate and its proportions between the study area, sample type, and breeds were presented using percentage. The association between the outcomes and variables such as breed, study area, and sample type was analyzed using the Fisher exact test. A *p* value ≤ 0.05 was considered statistically significant at 95% confidence level.

## 3. Results

### 3.1. Field Clinical Examination

**FIGURE 2:**
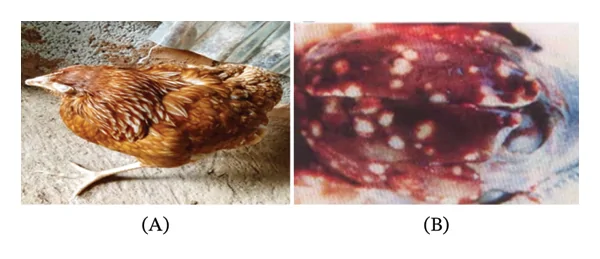
Clinical and postmortem manifestations of Marek’s disease virus in chickens. Source: antemortem and postmortem diagnosis conducted by the author during the study period. Arrows in (A) showing leg paralysis and blindness, in (B) enlargement of liver.

Field investigation conducted during MD outbreak revealed that the affected chickens displayed suggesting signs of the disease, including blindness, paralysis in their legs and wings, and a noticeable inappetence. When examining the dead chickens, we found swollen ischiatic nerves and distinctive white spots or tumor‐like growths on several organs, such as the liver, kidneys, heart, proventriculus, and spleen (Figure 2).

### 3.2. Real‐Time Polymerase Chain Reaction

**FIGURE 3:**
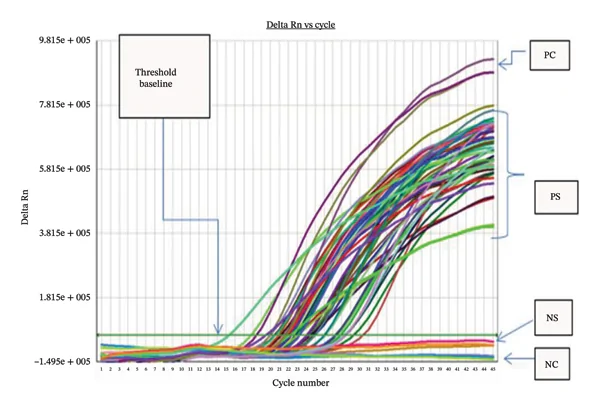
Amplification plot results of real‐time PCR for all samples. PS, positive samples; PC, positive controls; NS, negative samples; NC, negative controls.

Out of the 94 pooled samples tested using real‐time PCR to detect the Meq gene of MDV, nearly 80% (75 out of 94) tested positive for the MDV1 serotype. Of these, 36.17% (34/94) of the positive samples were obtained from feather follicles and the rest (43.62%; 41/94) were from postmortem tissue samples (Figure 3).

Out of 15 local breed chickens examined, 8 (53.3%) were positive for the virus, whereas 21 out of 23 exotic breed chickens (91.3%) tested positive. Overall, 29 out of 38 sampled chickens (76.31%) were positive by real‐time PCR. The difference in positivity between the two breed groups was statistically significant (Fisher = 9.01, *p* = 0.04) (Table 2).

**TABLE 2:** Comparison of real‐time PCR positivity between local and exotic chicken breeds.

	Level	No. of real‐time PCR positive	Fisher (*p* value)
Breed	Local	8/15 (53.3%)	
	Exotic	21/23 (91.3%)	9.01 (0.04)
	Total	**29/38 (76.31%)**	

*Note:* Bold values mean breed type.

MDV detection across districts was also determined based on the real‐time PCR assay. Accordingly, the highest proportion of MDV gene detection was observed in Holeta (95.65%), followed by the Mako District (88.89%) and Sebeta Subcity (62.5%). The least proportion was documented in Guto Gida District (57.89%). Significant differences were observed (*p* < 0.05) between the study areas (Table 3).

**TABLE 3:** Real‐time PCR and virus isolation findings across the investigated sites.

Areas	No. of pooled sample tested	Real‐time PCR positive		Virus isolation	
			Fisher (*p* value)		Fisher (*p* value)
Mako	36	32 (88.89%)		16 (50%)	
			10.32 (0.02)		

Guto Gida	19	11 (57.89%)		9 (81.81%)	
					11.10 (0.01)

Holeta	23	22 (95.65%)		10 (45.45%)	
Sebeta Subcity	16	10 (62.5%)		10 (100%)	

From 10 pooled liver samples, all samples (100%) tested positive for the virus using real‐time PCR. Similarly, both kidney (10/10 ;100%) and spleen (10/10, 100%) showed complete positivity. However, from six pooled samples of proventriculus samples, only one (1/6, 16.67%) sample was positive for the virus, whereas 34 (82.9%) pooled feather follicles from clinically sick chickens were positive for serotype 1 (MDV1) of the virus (Table 4).

**TABLE 4:** Comparison of real‐time PCR and virus isolation results across sample types.

Sample type	No. of pooled samples	Real‐time PCR positive		Virus isolation	
			Fisher (*p* value)		Fisher (*p* value)
Feather	41	34 (82.92%)		4 (11.76%)	
Ischiatic nerve	8	6 (75.00%)		6 (100%)	
Heart	9	4 (44.44%)	7.81 (0.19)	4 (100%)	11.03 (0.09)
Liver	10	10 (100%)		10 (100%)	
Kidney	10	10 (100%)		10 (100%)	
Spleen	10	10 (100%)		10 (100%)	
Proventriculus	6	1 (16.66%)		1 (100%)	
Total	94	75 (79.78%)		45 (60%)	

### 3.3. Isolation of MDV

**FIGURE 4:**
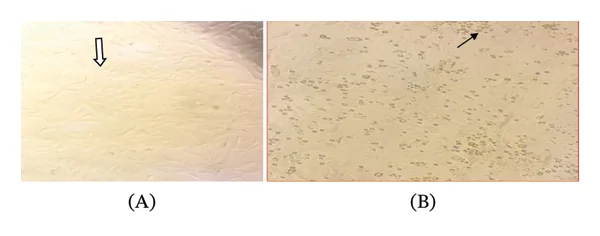
Cytopathic effect of MDV on the DF‐1 cells. The arrow in (A) indicates an uninfected cell; the arrow in (B) indicates small rounded and plaque cell.

All samples that tested positive by real‐time PCR were subsequently inoculated onto DF‐1 cell cultures for virus isolation and propagation. CPEs were observed in 45/75 (60%) of the samples. Initially, the CPE appeared as small, rounded cells. Later, it progressed to the formation of small plaques, with foci detaching from the surface of the culture flask. On the third passage, bright and enlarged cells were also observed under an inverted microscope (Figure 4). Conversely, no CPEs were observed in the remaining 30 samples (40%). Samples that did not show CPE were blind‐passaged up to third passage to declare negative against the test result.

## 4. Discussion

In the present study, MDV is detected and partially isolated in chickens showing clinical signs in Central and Western Ethiopia. Confirmation of the virus was achieved using molecular diagnostic techniques recommended by the World Organization for Animal Health [23].

In the present study, both clinical symptoms and postmortem findings indicate MD. This aligns with the previous studies conducted by Fenner et al. [24], Gopal et al. [25], Swath et al. [26], Suresh et al. [27], and Priathibha et al. [28]. The observed leg and wing paralysis is a hallmark symptom of MD, as the virus primarily affects the peripheral nervous system, leading to inflammation and dysfunction in nerves, particularly the sciatic nerve, which controls movement in the legs. Postmortem examination further supported the diagnosis by revealing lymphomas in critical organs such as the liver, kidney, heart, proventriculus, and spleen. This highlights the systemic nature of MD, which can cause solid tumors in multiple organs [18].

The overall detection rate of the MDV1 gene in this study was similar to earlier findings by Abdel et al. [29], Lopez‐Osorio et al. [30], and Rahman et al. [31], who reported detection rates of 77.6%, 70%, and 70%, respectively. However, it was higher than the rates reported by Abbas and Mehdi [32], Bayata et al. [33], and Duguma et al. [34], which ranged from 7.09% to 62.86%. Conversely, the detection rate in this study was lower than those reported by Davidson and Borenshtain [35], Yilmaz et al. [36], Mirtneh et al. [37], Demeke et al. [14], and Aman et al. [38], with rates ranging from 80% to 100%. These variations may be attributed to differences in management practices, ecological conditions, sample sizes, and types [13], as well as altitude differences reported by Kenenet [39] and Birhan [40].

Breed‐wise analysis in the present study indicated that exotic breeds showed significantly higher real‐time PCR positivity compared to local breeds. This finding is consistent with the report of Mastewal et al. [16] in Northwest Ethiopia. However, this observed difference in the current finding should be interpreted with caution because breed comparisons may be influenced by confounding factors such as vaccination status, management practices, and environmental conditions. Therefore, the higher PCR positivity observed in exotic breeds in this study may reflect differences in management, exposure risk, or vaccination history rather than inherent breed susceptibility. Previous studies have suggested that exotic chickens can be more vulnerable to diseases under certain environmental and management conditions due to lower adaptation to local stressors [13]. In addition, variation in sample size among breed groups might have contributed to the observed differences [38]. Further studies controlling for potential confounders such as vaccination status, management system, and flock biosecurity are required to clarify the relationship between breed and pathogen occurrence.

Significant variation in MDV gene detection was noted across different study areas, with the highest detection rate in the Holeta district. These differences could be due to varying management practices and chicken population densities, as densely populated flocks are at higher risk of disease transmission. MD spreads through airborne fomites and can survive on contaminated surfaces for over a year [41].

MDV1 gene detection from both feather follicles and postmortem tissues aligns with previous studies by Bulbula et al. [42], Lopez‐Osorio et al. [30], and Kenenet [39], who used qPCR to detect the virus. Among postmortem samples, the liver, kidneys, and spleen showed the highest detection rates, while the proventriculus had the lowest. The differential detection rates among postmortem tissues may reflect the tissue tropism of MDV1. This pattern aligns with the pathophysiology of MD, where lymphoproliferative lesions and viral replication are predominantly observed in visceral organs with high immune activity [43].

In this study, MDV replication was assessed by observing CPE characteristics in inoculated cells. Out of 75 samples inoculated on DF‐1 cells, 45 showed CPE typical of MDV, such as small, rounded cells, small plaques, with foci detaching from the surface of the culture flask and finally bright and enlarged cells, consistent with OIE [18]. This finding matches the reports of Heidari et al. [44] and Bulbula et al. [42] who observed similar CPE in DF‐1 and chicken embryo fibroblast cells. However, only 45 of the 75 qPCR positive samples showed CPE, while 30 did not, even after three passages. This could be attributed to several factors, including variability in the susceptibility of DF‐1 cells to different MDV strains or sample conditions or inhibitory substances or altered sample conditions that interfered with virus replication in vitro. OIE [18] also noted that the optimal culture system can affect virus viability.

## 5. Conclusion and Recommendation

This study offers valuable insights into the isolation and molecular detection of MDV in chickens from Central and Western Ethiopia. The findings shed light on the serious effects of the disease on poultry health and productivity in the region. The overall high prevalence of MDV among clinically suspected cases, with notable variation across regions, underscores the need for region‐specific interventions, including improved management practices, targeted vaccination programs, and enhanced surveillance to control the spread of MDV effectively. It also highlights the importance of understanding local risk factors to design tailored strategies for disease prevention and control. Virus isolation using DF‐1 cells showed CPEs in majority of samples, confirming active viral replication in a subset of cases. Detailed genetic characterization of the virus and differentiation of the vaccinal strain from the natural infection in exotic breeds are necessary to have effective prevention and control. Further experimental studies are needed to evaluate differences in susceptibility to MDV among local and exotic poultry breeds.

## Author Contributions

H.M.: data collection; Y.T.: investigation, supervising, and conceptualizing; B.S., A.A., and D.N.: laboratory analysis; D.A.: drafting; and A.K., B.B., and W.T.: data curation, analysis, and visualization.

## Funding

This study was supported by Wollega University, WU,202,431/26.

## Conflicts of Interest

The authors declare no conflicts of interest.

## Supporting Information

Additional supporting information can be found online in the Supporting Information section.

## Supporting information


**Supporting Information** Appendix I: Chicken Necropsy Protocols (Based on Corrie et al. [45] and Chauhan and Chandra [46]). The routine was reported to consist of six main steps. Step 1: Obtaining the History. Step 2: External Examination of the Bird. Step 3: Opening the Body. Step 4: Removal and Examination of Organs. Step 5: Examination and Sampling of Organs. Step 6: Writing the Report. Appendix II: DNA Extraction. It was described that a small feather was to be held with sterile forceps and that approximately 1 cm, divided into four pieces, was to be cut from the tip using sterile scissors. It was then indicated that 180 μL of buffer ATL and 20 μL of proteinase K should be added and that the mixture should be incubated at 56°C for 20 h. The mixture was to be vortexed repeatedly and briefly centrifuged. Subsequently, 200 μL of buffer AL was to be added, mixed for 15 s, and incubated for 10 min at 70°C. The suspension was then to be briefly centrifuged, after which 200 μL of molecular‐grade ethanol (96%–100%) was to be added and mixed for 15 s. The resulting mixture was to be applied to a QIAamp spin column. The column was then to be centrifuged for 1 min at 6000 × g (8000 rpm). The QIAamp spin column was subsequently to be placed in a clean collection tube, and the collection tube containing the filtrate was to be discarded. It was then indicated that the QIAamp spin column should be carefully opened and 500 μL of buffer AW1 added. The closed column was to be centrifuged for 1 min at 6000 × g (8000 rpm). The QIAamp spin column was then to be placed in a new collection tube, and the filtrate was to be discarded. Subsequently, 500 μL of buffer AW2 was to be added to the column. The tube was then to be closed and centrifuged for 3 min at 20,000 × g or 14,000 rpm. The QIAamp spin column was subsequently to be placed in a sterile reaction vessel, while the collection tube containing the filtrate was to be discarded. It was indicated that 200 μL of buffer AE should be pipetted into the QIAamp spin column and that the column should be incubated for 1 min  at room temperature. The column was then to be centrifuged for 1 min at 6000 × g or 8000 rpm. The filtrate was to be retained at 4°C for a maximum of 48 h, while prolonged storage was to be conducted at −20°C. Appendix III: PCR Mix (Master Mix) for MDV‐PCR. It was reported that the PCR master mix for MDV‐PCR consisted of 10 μL of Platinum PCR mix, 0.6 μL of forward primer MDV‐1F, 0.6 μL of reverse primer MDV‐1R, 0.8 μL of probe MDV‐1, 1 μL of BSA at 5 mg/mL, 2 μL of H_2_O, and 5 μL of sample DNA. The total reaction volume was reported to be 20 μL. Appendix IV: Reagents Used. It was reported that the reagents used included 180 μL of buffer ATL, 20 μL of proteinase K, 200 μL of buffer AL, ethanol at 96%–100%, 500 μL of buffer AW2, and 200 μL of buffer AE. Appendix V: Cell Culture. It was reported that tissue and feather follicle samples were initially chopped into small pieces using sterile scissors inside a Class II biosafety cabinet. The samples were then further ground into smaller pieces using a sterile mortar and pestle, with sand added to facilitate grinding, in the virology laboratory of the National Animal Health Diagnostic and Investigation Center. It was indicated that three to five samples collected from the same outbreak farm were pooled and processed to increase the likelihood of virus isolation. The samples were then transferred into sterile tubes and centrifuged for 10 min at 3000 rpm. The samples were subsequently filtered to remove debris and other contaminants. The supernatant was collected using a micropipette and transferred into a newly labelled tube. A 10% (w/v) suspension of the samples was reported to have been prepared in sterile PBS supplemented with penicillin at 100 IU/mL and streptomycin at 1000 μg/mL. The suspension was then transferred into a sterile tube and centrifuged at 3000 rpm for 10 min. The supernatant was collected and inoculated onto confluent primary Douglas Foster‐1 (DF‐1) cell maintained in GMEM containing 2% bovine fetal calf serum and incubated at 37°C. It was reported that the cultures were observed daily using an inverted microscope for up to 1 week to determine the presence of cytopathic effects (CPEs) characteristic of MDV. Samples that did not show CPE were reported to have been blindly passaged up to the third passage. Samples that did not develop CPE by the third blind passage were considered negative, whereas samples that demonstrated characteristic CPE were considered positive and were kept at −20°C for further analysis.

## Data Availability

The data used to support the findings of the study are available from the corresponding author (Y.T.) upon request.

## Appendix 1 Study Protocol

Project title: Outbreak Investigation of Marek’s Disease in Chickens in Central and Western Ethiopia.

Institutional affiliation: Wollega University, School of Veterinary Medicine.

Ethical approval number: Vetm 21/8/2022‐25/8/2022.

Approving body: Animal Welfare and Research Ethics Review Committee (AWRERC), Wollega University.

1. Objective and study scope
The primary objective of this protocol was to investigate suspected outbreaks of Marek’s disease in poultry flocks across selected districts of Central and Western Ethiopia. The study intended to confirm the virus through clinical, gross pathological, and molecular diagnostic methods.
2. Animal welfare and live animal handling exclusion
  • Live animal involvement: This protocol involved zero (0) interventions, handling, housing, or monitoring of live animals.
  • Exclusion of welfare checks: Because no live animals were manipulated by the research team, standard animal welfare monitoring procedures (such as routine health checks, temperature taking, or weight monitoring) were not applicable to this study design.
  • Inclusion criteria: Sampling was strictly restricted to chickens that had been infected naturally by suspected disease within a maximum window of 12 h prior to the team’s arrival. No live animals were euthanized or harmed for the purpose of this research.
3. Owner consent and ethical compliance
  • Prior to sample collection, the investigative team provided the flock owners with a clear explanation of the study’s purpose.
  • Formal permission/consent was obtained from each flock owner before conducting postmortem examinations on their deceased poultry.
  • All procedures adhered strictly to institutional, national, and international guidelines for ethical veterinary research.
4. Field postmortem and tissue sampling procedures
  • Field necropsies were performed using standard personal protective equipment (PPE) and strict biosecurity measures to prevent the mechanical transmission of pathogens between farms.
  • Gross pathology: Thorough postmortem examinations were conducted on the deceased chickens to observe and record pathognomonic lesions of Marek’s disease (e.g., enlargement of peripheral nerves and visceral tumors in organs).
  • Sample collection: Relevant tissue samples (e.g., sciatic nerves, spleen, liver, and feather follicles) were aseptically collected using sterile instruments.
  • Preservation and transport: Samples for molecular analysis were stored in tube containing viral transport media and transported immediately via cold chain to the virology and molecular laboratory at the Animal Health Institute, Sebeta, Ethiopia, for further processing.
5. Data de‐identification and confidentiality
To protect the rights and privacy of the participating poultry farmers, all farm identifiers, owner names, and precise geographic coordinates were de‐identified and coded numerically during data analysis and reporting.

## References

[1] Davison F. and Kaiser P. , Davison F. and Nair V. , Immunity to Marek’s Disease, Marek’s Disease: An Evolving Problem, 2004, Elsevier Academic Press, London, 126–141.

[2] Ewie S. S. , Mady W. H. , Hamad E. M. , Arafa A. A. , Tamam S. M. , and Madbouly H. M. , Isolation and Molecular Characterization of Marek’s Disease Virus from Layers Chicken in Egypt, Journal of Veterinary Medicine and Research. (2021) 27, no. 2, 168–177.

[3] Jenberie S. , Lynch S. E. , Kebede F. et al., Genetic Characterization of Infectious Bursal Disease Virus Isolates in Ethiopia, Acta Tropica. (2014) 130, 39–43, 10.1016/j.actatropica.2013.09.025. PMC4008939 24145155

[4] Davis M. F. and Morishita T. Y. , Poultry Necropsy Basics. Extension Fact Sheet, 2001, 12, The Ohio State University.

[5] Duguma R. , Dana N. , and Yami A. , Marek’s Disease Vaccination Opened the Door to Rear Indigenous Chickens of Ethiopia Under Confined Management, International Journal of Applied Research in Veterinary Medicine. (2006) 4, no. 2, 121–127.

[6] Atkins K. E. , Read A. F. , Walkden-Brown S. W. , Savill N. J. , and Woolhouse M. E. , The Effectiveness of Mass Vaccination on Marek’s Disease Virus (MDV) Outbreaks and Detection Within a Broiler Barn: a Modeling Study, Epidemics. (2013) 5, no. 4, 208–217, 10.1016/j.epidem.2013.10.001. PMC3863959 24267877

[7] McPherson M. and Delany M. , Virus and Host Genomic, Molecular, and Cellular Interactions During Marek’s Disease Pathogenesis and Oncogenesis, Poultry Science. (2016) 95, no. 2, 412–429, 10.3382/ps/pev369. PMC4957504 26755654

[8] Rozins C. , Day T. , and Greenhalgh S. , Managing Marek’s Disease in the Egg Industry, Epidemics. (2019) 27, 52–58, 10.1016/j.epidem.2019.01.004. 30745241

[9] Puro K. U. , Bhattacharjee U. , Baruah S. et al., Characterization of Marek’s Disease Virus and Phylogenetic Analyses of Meq Gene from an Outbreak in Poultry in Meghalaya of Northeast India, VirusDisease. (2018) 29, no. 2, 167–172, 10.1007/s13337-018-0448-2. PMC6003054 29911149

[10] Wen Y. , Huang Q. , Yang C. et al., Characterizing the Histopathology of Natural Co-Infection With Marek’s Disease Virus and Subgroup J Avian leucosis Virus in Egg-Laying Hens, Avian Pathology. (2018) 47, no. 1, 83–89, 10.1080/03079457.2017.1375079. 28859493

[11] Cadmus K. J. , Mete A. , Harris M. et al., Causes of Mortality in Backyard Poultry in Eight States in the United States, Journal of Veterinary Diagnostic Investigation. (2019) 31, no. 3, 318–326, 10.1177/1040638719848718. PMC6838705 31084344

[12] Neerukonda S. N. , Taylarides-Hontz P. , MacCarthy F. , Pendarvis K. , and Parcells M. S. , Comparison of the Transcriptomes and Proteomes of Serum Exosomes from Marek’s Disease Virus Vaccinated and Protected and Lymphoma-Bearing Chickens, Genes. (2019) 10, no. 2, 10.3390/genes10020116. PMC6410298 30764491

[13] Duguma R. , Yami A. , Dana N. , Hassen F. , and Esatu W. , Marek’s Disease in Local Chicken Strains of Ethiopia Reared Under Confined Management Regime in Central Ethiopia, Revue de Medecine Veterinaire. (2005) 156, no. 11.

[14] Demeke B. , Jenberie S. , Tesfaye B. et al., Investigation of Marek’s Disease Virus from Chickens in Central Ethiopia, Tropical Animal Health and Production. (2017) 49, no. 2, 403–408, 10.1007/s11250-016-1208-1. 27975190

[15] Abdela B. , Bizunesh B. , Biniam T. , Abde A. , and Demessa N. , Isolation and Molecular Detection of Marek’s Disease Virus from Outbreak Cases in Chicken in South Western Ethiopia, Veterinary Medicine: Research and Reports. (2022) 13, 265–275. 10.2147/VMRR.S376795 PMC9527818 36199365

[16] Mastewal B. , Nega B. , Molalegne B. et al., Sero-Epidemiology of Marek’s Disease Virus on Local and Exotic Chickens in Northwest Ethiopia, Journal of World’s Poultry Research. (2021) 11, no. 1, 53–63.

[17] Kassahun B. , Wudu T. , Animaw S. et al., Seroprevalence and Risk Factor Modeling of Marek’s Disease Virus in Indigenous Tilili Breed Chickens of the Amhara Region, Ethiopia, Comparative Immunology, Microbiology and Infectious Diseases. (2025) 120. 10.1016/j.cimid.2025.102341 40319574

[18] OIE , Manual of Diagnostic and Vaccine for Terrestrial Animals, World Health Organization for Animal Health. Version Adopted by the World Assembly of Delegates of the OIE. (2018) 16–18.

[19] Gall S. , Kőrösi L. , Cortes A. et al., Use of Real-Time PCR to Rule Out Marek’s Disease in the Diagnosis of Peripheral Neuropathy, Avian Pathology. (2018) 47, no. 4, 427–433, 10.1080/03079457.2018.1473555. 29745244

[20] Dunn J. , Southard T. , Cooper C. , Kiupel M. , and Witter R. L. , Diagnosis of Marek’s Disease From a Japanese Quail (Coturnix Japonica) Using Paraffin-Embedded Liver [Abstract], American Association of Avian Pathologists Symposium and Scientific Program, 2010.

[21] Freshney R. I. , Culture of Animal Cells: A Manual of Basic Technique and Specialized Applications, 2010, 6th edition, John Wiley & Sons Ltd, Hoboken.

[22] R Core Team , R: A Language and Environment for Statistical Computing, 2017, R Foundation forStatistical Computing, Vienna, Austria.

[23] Woah , WOAH Manual of Diagnostic Tests and Vaccines of Terrestrial Animals, Marek’s Disease. Paris, France: Office International des Epizootics. (2010) 2010, 1–11.

[24] Fenner S. , Richard W. , Ronald A. et al., Veterinary Virology, 2011, 4th edition, Academic Press, Elsevier, London, UK, 180–193.

[25] Gopal S. , Manoharan P. , Kathaperumal K. , Chidambaram B. , and Divya K. C. , Differential Detection of Avian Oncogenic Viruses in Poultry Layer Farms and Turkeys by Use of Multiplex PCR, Journal of Clinical Microbiology. (2012) 50, no. 8, 2668–2673, 10.1128/JCM.00457-12. PMC3421529 22675132

[26] Swathi B. , Kumar A. , and Reddy M. R. , Histological and Molecular Diagnosis of Poultry Tumours, Indian Journal of Veterinary Pathology. (2012) 36, no. 1, 41–48.

[27] Suresh P. , Johnson Rajeswar J. , Sukumar K. , Harikrishnan T. J. , and Srinivasan P. , Pathotyping of Recent Indian Field Isolates of Marek’s Disease Virus Serotype 1, Acta Virologica. (2015) 59, no. 02, 156–165, 10.4149/av_2015_02_156. 26104332

[28] Priathibha Y. , Sreedevi B. , Kunar N. V. , and Srilatha C. H. , Molecular Characterization and Phylogenetic Analysis of Oncogenes From Virulent Serotype-1 Marek’s Disease Virus in India, Acta Virologica. (2018) 62, 277–286. 10.4149/av_2018_221 30160143

[29] Abd-Ellatieff H. A. , Rawash A. A. , Ellakany H. , Goda W. M. , Suzuki T. , and Yanai T. , Molecular Characterization and Phylogenetic Analysis of a Virulent Marek’s Disease Virus Field Strain in Broiler Chickens in Japan, Avian Pathology. (2018) 47, 47–57. 10.1080/03079457.2017.1362497 28762757

[30] Lopez-Osorio S. , Villar P. , Peidrahita G. et al., Molecular Detection of Marek’s Disease Virus in Feather and Blood Samples from Young Laying Hens in Colombia, Acta Virologica. (2019) 63, no. 4, 380–391, 10.4149/av_2019_402. 31802681

[31] Rahman M. W. , Nooruzzaman M. , Suma U. S. , Chowdhury E. H. , and Islam M. R. , Comparative Suitability of the Feathers, Peripheral Blood and Spleen Tissue Samples of Chickens for the Detection of Marek’s Disease Virus by Polymerase Chain Reaction, Bangladesh Veterinarian. (2019) 35, no. 1–2, 1–6, 10.3329/bvet.v35i1-2.53381.

[32] Abbas D. and Mehdi G. , Molecular Study for Detection of Marek′s Disease Virus in South West of Iran, Scientific Research and Essays. (2011) 6, no. 12, 2560–2563.

[33] Bayata S. , Abde A. , and Melaku S. , Molecular Detection from Chicken’s Feather in Central Ethiopia, Life Science Journal. (2021) 18, no. 12, 18–21.

[34] Duguma R. , Yami A. , Dana N. , Hassen F. , and Esatu W. , Marek’s Disease in Local Chicken Strains of Ethiopia Reared Under Confined Management Regime in Central Ethiopia, Revue de Medecine Veterinaire, 2005, 11, Toulouse, France.

[35] Davidson I. , Borenshtain R. , and Weisman Y. , Molecular Identification of the Marek’s Disease Virus Vaccine Strain CVI988 in Vaccinated Chickens, Journal of Veterinary Medicine Series B. (2002) 49, 83–87. 10.1046/j.1439-0450.2002.00512.x 12002424

[36] Yilmaz A. , Turan N. , Bayraktar E. et al., Molecular Characterization and Phylogenetic Analysis of Marek’s Disease Virus in Turkish Layer Chickens, British Poultry Science. (2020) 61, no. 5, 523–530, 10.1080/00071668.2020.1758301. 32316760

[37] Mirtneh A. , Isolation and Molecular Characterization of Marek’s Disease Virus in Central Ethiopia and Evaluation of Its Vaccine Trial, 2015, Addis Ababa University, College of Veterinary Medicine and Agriculture, Debre Zeit, Ethiopia, 18–24, MSc thesis.

[38] Aman K. , Maan N. S. , Mahajan N. K. , Kanisht B. , Ranjan K. , and Sushila M. , Isolation and Molecular Characterization of MDV-1 From Vaccinated Poultry Birds in Haryana State of India. Proceedings of the National Academy of Sciences, India, Section B, Biological Science. (2022) 92, no. 3, 679–690.

[39] Kenenet A. , Isolation and Molecular Characterization of Chicken Marek′s Disease Virus in Selected Districts of Ethiopia, 2017, Addis Ababa University, College of Veterinary Medicine and Agriculture, Department of Microbiology, Immunology and Veterinary Public Health, 23–28, MSc thesis.

[40] Birhan D. , Shiferaw J. , Biruk T. et al., Investigation of Marek′s Disease Virus From Chickens in Central Ethiopia, Tropical Animal Health and Production. (2016) 49, 403–408. 10.1007/s11250-016-1208-1 27975190

[41] Schat K. and Nair V. , Marek’s Disease Virus, Diseases of Poultry, 2008, 12th edition, Iowa State Press, Ames, Iowa, 449–514.

[42] Bulbula A. , Bizunesh B. , Biniam T. , Abde A. , and Demessa N. , Isolation and Molecular Detection of Marek’s Disease Virus From Outbreak Cases in Chicken in South Western Ethiopia, Veterinary Medicine: Research and Reports. (2022) 13, 265–275, 10.2147/vmrr.s376795. PMC9527818 36199365

[43] Ahmed H. , Mays J. , Kiupel M. , and Dunn J. R. , Development of Reliable Techniques for the Differential Diagnosis of Avian Tumour Viruses by Immunohistochemistry and Polymerase Chain Reaction from Formalin Fixed Paraffin Embedded Tissue Sections, Avian Pathology. (2018) 47, no. 4, 364–374, 10.1080/03079457.2018.1451620. 29533078

[44] Heidari M. , Wang D. , Fitzgerald S. D. , and Sun S. , Severe Necrotic Dermatitis in the Combs of Line 63 Chickens Infected with Marek’s Disease Virus, Avian Pathology. (2016) 45, no. 5, 582–592, 10.1080/03079457.2016.1189511. 27215315

[45] Nair V. , Gimeno I. , Dunn J. et al., Swayne D. E. , Boulianne M. , Logue C. M. , McDougald L. R. , Nair V. , and Suarez D. L. , Neoplastic Diseases, Diseases of Poultry, 14th, 2019, Wiley, 550–587.

[46] Chauhan R. S. and Chandra D. , Chauhan R. S. and Chandra D. , Necropsy and Histopathological Examination Techniques, Veterinary Laboratory Diagnosis, 2007, Indian Veterinary Research Institute, 243–269.

