# Supplementary material for: Outbreak Investigation of Marek’s Disease in Chickens in Central and Western Ethiopia
Source: Vet Med Int. 2026 Sep 21;2026:1760044. doi: 10.1155/vmi/1760044 (PMC13591230; doi:10.1155/vmi/1760044)
Supplement: Supplementary file 1 [file VMI-2026-1760044-s001.docx]

**Appendix I**

Chicken’s Necropsy Protocols (Corrie *et al*. (2008) and Chauhan and Chandra. (2007)

A good necropsy involves paying attention to ALL the clues that can be provided, so the routine has to be followed, with attention to detail at every step. The consistent routine entails 6 steps:

**Step 1:** Obtain the History ; this history should include:

- Bird’s age
- Sex
- Breed
- Clinical signs
- History of trauma or disease
- History of any treatments administered
- Any other information that may be relevant to the case such as type of feed and water
- If the bird is a member of a flock the following is also required:
- Number of birds in the flock
- Number of birds in the affected group
- Number of affected birds
- Clinical signs of the flock should also be noted

**Step 2:** Examine the Bird Externally

- Examine the bird for any signs of trauma and evaluate the bird’s general body condition.
- If possible the bird should be weighed.
- Prior to opening the body:
- If it is planned for additional investigation, you might want to swab the trachea or cloaca. When swabbing the trachea, insert the swab up in the choana
- Dislocate cervical joints of the bird and dip the whole bird/carcass into a bucket of soapy water to thoroughly wet all the feathers.

**Step 3**: Open the Body

- The body should be placed on its back with its feet facing you and reflect the wings back.
- Cut through the skin between the legs and the breast so the legs can be fully abducted and lie flat against the table.
- Remove the skin from the ventral surface of the bird by cutting across at the caudal edge of the keel and then pulling skin cranially and caudally - peel away from the muscle to expose the muscular body wall.
- Make a small cut into the body cavity using scissors or a scalpel blade just behind the breast bone, and then pull the abdominal muscle caudally to expose some abdominal viscera.
- Extend the cut up through the cervical area and cut open the beak at the angle of the jaw.
- Now oral cavity, oesophagus, trachea and crop are all visible
- The keel bone and breast muscle is then removed by incising the pectoral muscles on each side of the keel and cutting through the ribs.
- Use the heavy poultry shears. Remove the keel and breast muscles entirely - you should now be able to see the internal organs from oral cavity to rectum.
- At this point, all internal viscera should be examined in situ for any abnormalities before removing any organs.
- Note the color, position and size of all organs and look for any adhesions.
- Examine the air sacs for increased thickness or cloudiness (caudal thoracic air sac is at the end of the forceps).

**Step 4:** Remove the Organs and Set Aside For Examination

- It is probably easiest to remove the abdominal viscera first and then go back and remove the thoracic organs. Remove the liver - in birds the liver takes up a big portion of the abdomen.
- The spleen can be a difficult organ to find once everything is removed so it is a good idea to locate it now and set it aside.
- Pull the proventriculus aside and it should pop into view. Take it out now and set it in a clean dry spot.
- Find the junction of the oesophagus and stomach, cut here and pull the digestive tract out, all the way to the cloaca.
- Cut at the end of the large intestine, but leave the bursa in the bird.
- Kidneys are nestled up against the body wall - there are three portions - cranial, middle and caudal poles.
- In females only the left side of the tract persists, the right is vestigial and too small to be identifiable.
- Pull the heart and lungs away - the lungs are closely adhered to the dorsal body wall, and therefore, careful teasing of the lungs away from the ribs may be necessary to remove them.
- Open up the nasal cavity to take a close look at the sinuses.
- Observe the sciatic nerve which is an important location where Marek’s disease can be seen.
- Open some joints to observe the fluid and synovial membranes.
- The brain can now be removed. Using the smaller scissors (not poultry shears), chip away at the skull, beginning from the foramen magnum, and remove the calvarium covering the cerebral hemispheres and the cerebellum. Take the brain out - use care this organ is quite soft.

**Step 5**: Examine and Sample the Organs

A) Examining of the organs

- It is a good idea to go from the “cleanest” organs to the “dirtiest. Usually this order is: lymphoid tissue, brain, lungs, heart, kidneys, reproductive tract, liver, intestinal tract.
- Note any abnormalities for each (color, size, shape, consistency, exudates). Be sure to examine both capsular and cut surface.
- Make several cuts in each organ and collect specimens for further diagnostic work.

B) Sampling of the Organs

- Tissues should be collected in duplicate, with half going into 10% formalin for histopathology and half as unfixed tissues that will be used for bacterial culture etc. if necessary.
- The following tissues should be collected in all cases: lung, liver, spleen, bursa, brain, kidney, intestines.
- Additionally, any other tissue that might have lesions (skin, adrenal, ovary, heart etc.) should also be collected.
- Label the tissues that are collected fresh so that the technician performing the tests can determine what each tissue is.
- Crushing should be avoided by using a sharp blade and a hard surface when taking samples since this may cause artefact histologically.
- When examining any organ with a mucosal surface (trachea, oesophagus, intestine, etc.) care should be taken not to damage or destroy the mucosal surface by rubbing the surface with fingers or instruments.
- In order to improve fixation of tissues, samples should not exceed 5mm in thickness and volume of fixative should be at least 10 times the volume of tissue.
- Fresh samples should be packaged so that they remain cool and to minimize possibilities of leaking.

**Step 6**: Write the Report

- No necropsy is complete until all findings have been recorded in written form.
- The report should include at least the following information:
- Species, breed, age, sex and history
- Dead or euthanized?
- Nutritional, hydration status
- Findings from external examination
- Findings by organ system: Lymphoreticular (spleen, bursa), Respiratory, Digestive, Urogenital, Musculoskeletal, Nervous.

**Notes:** When describing tissues, keep these four qualities in mind - consistency, color, size, and symmetry.

**Appendix II**

**DNA extraction**

Hold a small spring with sterile forceps, and then cut with a sterile scissors 1 cm (4 pieces) from the tip of spring off on.

Pipette 180 µl in this buffer ATL and 20 µl proteinase K and incubate at 56 0 c for 20 hours.

Vortex repeatedly and centrifuge briefly 62 Add 200 µl buffer Al mix for 15 seconds and incubate the mixture for 10 minutes at 70 0 c.

Centrifuge briefly Centrifuge the suspension briefly with centrifuge tubes and add 200 µl of molecular grade Ethanol (96-100%) and mix for 15 seconds Apply this mixture to precipitate in to a QIAamp spin column

The column is then centrifuge for 1 minute at 6000g(8000 rpm)

Place the QIAamp spin column in a clean collection tube.

Discard the collection tube with filtrate.

Carefully open to the QIAamp spin column and add 500 µl buffer AW1.

The closed column is then centrifuge for 1 minute at 6000g (8000 rpm).

Place QIAamp spin column in a new collection tube and discard the filtrate Carefully open to the QIAamp spin column and add 500 µl buffer AW2.

close the tube and centrifuge for 3 minutes 20000g or 14,000 rpm.

Place QIAamp spin column in a sterile reaction vessel and discard the collection tube with filtrate.

Pipette 200 µl of buffer AE in the QIAamp spin column.

Perform a 1 minute incubation at room temperature Centrifuge for 1 minute at 6000g or 8000rpm and retain the filtrate at 4 o c for 48 hours at the latest.

Prolonged storage at -20o c .

**Appendix III**

PCR Mix (Master Mix.) for MDV-PCR

Reagents	Volume of (µl) final concentration

Platinum PCR mix	10

Primer forward MDV-1F	0.6

Primer reverse MDV-1R	0.6

Probe MDV-1	0.8

BSA 5mg.ml-1	1

H2O	2

Sample DNA	5

Total volume (µl) 20

**Appendix IV**

Reagents used

180 µl of buffer ATL

20 µl of proteinase K

200 µl buffer Al

Ethanol (96-100%)

500 µl of buffer AW2

200 µl of buffer AE

**Appendix V**

**Cell culture**

Tissue and Feather follicle samples was first chopped into small pieces using sterile scissor inside the Biosafety cabinet class II and further ground in to smaller pieces using sterile mortar and pestle by adding sand to facilitate grading in the virology laboratory of the National Animal health Diagnostic and investigation Center. Three to five samples collected from the same outbreak farm was pooled and processed to increase the chance of virus isolation.

Then the samples were added to the sterile tube and centrifuged for 10 min at 3000rpm. Then the samples were filtered to remove debris and other contaminants and the supernatant was harvested by micropipette and add to the new labeled tube. A 10% (w/v) suspension of samples was prepared in sterile phosphate buffer saline (PBS) supplemented with penicillin (100 IU/ml) and streptomycin (1000 μg/ml). The suspension was transferred into sterile tube and centrifuged at 3000 rpm, for 10 minutes. Supernatants was harvested and inoculated onto confluent primary Duck Forster cells in maintenance GMEM containing 2% bovine foetal calf serum and incubated at 37ºC.

Cultures were observed daily using inverted microscope for up to 1 week to check for the presence of cytopathic effect (CPE) characteristic for MDV. Those that had not shown CPE was blindly passaged up to the third passage. Samples which did not develop any CPE until the third blind passage were considered as negative, whereas samples revealing characteristics CPE was considered as positive and kept at -20ºC for further analysis
